# Vitamin D Deficiency Trends, Risk Factors, and Occupational Risk in Active Component Service Members of the U.S. Armed Forces, 2018–2022

**Published:** 2024-08-20

**Authors:** Devin C. Kelly, Michael Fan, Richard S. Langton, Shauna L. Stahlman

**Affiliations:** 1 Uniformed Services University of the Health Sciences, Department of Preventive Medicine and Biostatistics, Bethesda, MD; 2 Armed Forces Health Surveillance Division, Defense Health Agency, Silver Spring, MD

## Abstract

Vitamin D contains 2 related fat-soluble substances, D3 and D2, that are essential for bone health and overall well-being. The burden of vitamin D deficiency within the active component of the armed forces is unknown. This study describes trends of vitamin D deficiency diagnoses in the active component of the U.S. Armed Forces. Risk factors for vitamin D, such as military occupation, were examined to see if preventive measures and targeted vitamin D screening would be beneficial, as the United States Preventive Task Force does not recommend universal screening for vitamin D, nor does TRICARE cover screening for asymptomatic individuals. The surveillance period covered January 1, 2018 through December 31, 2022. The data were derived from the Defense Medical Surveillance System (DMSS). Vitamin D deficiency was measured using ICD-9-CM and ICD-10-CM diagnoses recorded in inpatient and outpatient medical encounters. Incidence rate and average annual prevalence were calculated. A logistic regression was performed to obtain adjusted odds ratios. The rates of vitamin D deficiency diagnoses among active component service members (ACSMs) remained steady during the study period, with an incidence rate of 16.4 per 1,000 person-years and an average annual prevalence of 2.2%. Female service members, those of older age groups, and indoor workers demonstrated higher rates of vitamin D deficiency. Previously described demographic risk factors such as indoor work and history of obesity or malabsorption syndrome were also associated in this study with vitamin D deficiency in ACSMs, although older age groups in this study were not associated with vitamin D deficiency. Pilots and air crew had the lowest rates of vitamin D deficiency, while health care workers had the highest, when evaluating by occupation.

**What are the new findings?:**

Throughout the study's 2018-2022 period of surveillance, the rates of vitamin D deficiency among active component service members remained steady, with an overall incidence rate of 16.4 per 1,000 person-years and a total average annual prevalence of 2.2%. Female sex, older age, and indoor workers had higher rates of vitamin D deficiency.

**What is the impact on readiness and force health protection?:**

Understanding the trends and risk factors for vitamin D deficiency in active component service members can inform policy that will affect populations that could benefit from education on vitamin D deficiency and prevention, as well as informing clinicians about individuals at risk for vitamin D deficiency. Treatment of vitamin D deficiency may increase physical performance, reduce risk of fractures, and contribute to overall health. Adequate vitamin D levels in the force may increase mission and duty availability.

## 1 BACKGROUND

Vitamin D contains 2 related fat-soluble substances, cholecalciferol (D3) and ergocalciferol (D2), that are essential for bone health and overall well-being.^1,2^ Vitamin D deficiency is defined by having a serum 25(OH)D concentration under 50 nmol/liter.^3^ Sufficient vitamin D levels in athletes have correlated with better physical performance, increased power, strength, and VO2 max.^4^ Deleterious health effects in adults with vitamin D deficiency include increased risk for fractures, muscle weakness, and metabolic bone disease.^1,3,5^ In contrast, several studies have highlighted the potential health benefits of adequate vitamin D levels, which can be acquired through diet, dietary supplements, or sun exposure. In particular, those taking daily or weekly vitamin D supplementation have been shown to have lower odds of developing acute respiratory infections.^6^

Vitamin D deficiency is an important consideration for military readiness because of the association with increased risk of infections and injury and worse physical performance, leading to reduced training time and mission availability. Some studies, for example, have highlighted risks to bone health in recruited trainees. Stress fractures are more likely in basic military trainees with low vitamin D levels.^7,8,9^ In female Navy recruits, calcium and vitamin D supplementation reduced incidence of stress fractures.^10^ Vitamin D deficiency may play a role in chronic illnesses such as cancers, autoimmune diseases, and cardiovascular disease.^5^ Understanding the trends and risk factors for vitamin D deficiency can help identify populations that may benefit from education and interventions to address vitamin D deficiency in active component service members (ACSMs).

The incidence and prevalence of vitamin D deficiency among U.S. ACSMs and any potential risk factors have not been described. The first objective of this study was to describe the trends of vitamin D deficiency in the active component in the past 5 years. The second objective was to identify factors independently associated with a current vitamin D deficiency diagnosis, with particular emphasis on the occupation category. Occupation was a focus because indoor occupations, such as shift workers, health care workers, and submariners, have a higher risks of vitamin D deficiency than outdoor workers, presumably due to less sunlight exposure.^11,12^

## 2 METHODS

The surveillance period covered January 1, 2018 through December 31, 2022. The surveillance population included all ACSMs of the U.S. Army, Navy, Air Force, and Marine Corps. The data used to determine incident cases of vitamin D deficiency were derived from the Defense Medical Surveillance System (DMSS), which documents both ambulatory encounters and hospitalizations of ACSMs of the U.S. Armed Forces in fixed military and civilian (if reimbursed through the Military Health System) hospitals and clinics. Periodic Health Assessment (PHA) data have been captured by DMSS since 2018.

Cases of vitamin D deficiency were defined by retrieving diagnostic codes (ICD-9: 268.9 or 268.2, ICD-10: E55.9) in any diagnostic position from the outpatient, inpatient, or Theater Medical Data Store (TMDS). For the incidence analysis, the incident date was defined as the date of the first medical encounter that included a defining diagnosis of vitamin D deficiency. Any ACSM diagnosed with vitamin D deficiency before 2018 was excluded from the incidence analysis, and person-time was censored at the incident date. Aggregated person-years (p-yrs) of service was used as the denominator. For the prevalence analysis, cases were counted each year with an outpatient, inpatient, or TMDS medical encounter with a vitamin D diagnosis in any diagnostic position. One prevalent case was counted per person per year. The mid-year ACSM population was the denominator for calculating average annual prevalence.

Covariates in this analysis included basic demographics, geographic latitude of military unit assignment, obesity, history of malabsorption syndrome, self-reported dietary factors, and vitamin supplementation. Covariates were chosen based on the known association with vitamin D deficiency.^3,5,11,13,14,15,16,17,18^ Countries, states, and ZIP codes (when applicable) of the military unit assignment were divided according to locations at or below 33° and above 33°. Obesity was categorized into ‘Yes’ or ‘No’ through a combination of ICD-10 codes and PHA height and weight data. Height and weight data from the PHA were used to calculate BMI, and anyone with a BMI of 30 or greater was classified as having obesity for the year of their weight measurement. In addition, if an individual had an outpatient encounter with an obesity diagnosis (ICD-10: Z683*, Z684*, E660*, E661, E662, E668, or E669), the person was classified as having obesity during that year of diagnosis; otherwise, individuals were categorized as not being obese. Malabsorption syndrome was defined by a prior diagnosis of Crohn’s disease, ulcerative colitis, or other type of intestinal malabsorption syndrome (ICD-9: 555*, 556*, and 579*, ICD-10: K50*, K51*, and K90*). Department of Defense Duty Military Occupation Specialty (DMOS) codes were organized into indoor and outdoor occupations.

Dietary factors and multivitamin supplementation were derived from PHA responses. Nutritional factors included frequency of consumption of dairy, calcium-containing foods, and fish within the past 30 days. These factors were chosen because they are known sources of vitamins D2 and D3.^3^ The frequency of multivitamin supplementation within the past 12 months (or since the last PHA) was measured. Information about vitamin D supplementation (within the past 12 months) was unavailable until the August 2021 version of the PHA form; therefore, these data were only analyzed for calendar year 2022. Responses to these questions were categorized according to the frequency expected to satisfy vitamin D dietary requirements by the Endocrine Society^3^ or current USDA recommendations.^19^ For individuals missing a PHA in a given year, responses were imputed from the subsequent or prior year when available; otherwise, responses were left as unknown/missing.

In the secondary analysis, logistic regression was used to calculate the adjusted odds of being diagnosed as a prevalent case in 2022. The independent variables included in the model were sex, age, race and ethnicity, service branch, military unit latitude, obesity, history of malabsorption syndrome, and primary occupation category.

## 3 RESULTS

The 104,994 incident cases of vitamin D deficiency diagnoses among ACSMs during the 2018-2022 surveillance period resulted in an overall incidence rate of 16.4 cases per 1,000 p-yrs. The total average annual prevalence of vitamin D deficiency diagnosis among ACSMs during the surveillance period was 2.2%. Incidence rates and average annual prevalence remained steady throughout the study period. The incidence and prevalence peaked during 2021 at 18.3 per 1,000 p-yrs and 2.4%, respectively (**Figure**). Rates among all categories remained consistent during the surveillance period.

Crude (i.e., unadjusted) incidence rates and prevalence by demographic categories are shown in **Table 1**. The total incidence rate and average annual prevalence were more than 2 times higher among women than men. The rates of vitamin D deficiency increased in those aged 30-39 years compared to those aged 20-29 years and less than age 20 years and were highest in those over age 40 years. Among racial and ethnic groups, rates of vitamin D deficiency were higher for persons other than non-Hispanic Whites. Recruits had the highest vitamin D deficiency diagnosis rates compared to enlisted personnel and officers. The Marine Corps had the lowest vitamin D deficiency diagnosis rates among the service branches. Rates were higher in those assigned to a military unit located above 33° latitude. Those with obesity and a history of malabsorption syndrome had higher rates than those without. Those taking multivitamins and vitamin D supplementation had higher rates than those not using vitamin D supplementation. The incidence rate in those taking vitamin D supplementation more than once a week and once a week or less often was 52.0 and 15.5 per 1,000 p-yrs, respectively. Pilots and air crew had the lowest rates when evaluated by primary occupational category, while health care occupations had the highest rates. Those with an indoor occupation had more than double the rates of vitamin D deficiency than those with an outdoor occupation.

In the logistic regression model, pilots and air crew had the lowest odds of vitamin D deficiency compared to other occupations (adjusted odds ratio=0.52, 95% confidence interval=0.47, 0.58) (**Table 2**). Service members in the active component who were female, of older age, non-Hispanic Black race and ethnicity, at geographic latitude above 33°, obese, and with history of malabsorption syndrome had higher odds of being diagnosed with vitamin D deficiency compared to their respective reference groups. Among the service branches, the Marine Corps had the lowest vitamin D deficiency diagnosis odds.

## 4 DISCUSSION

The results of this study show a steady trend of vitamin D deficiency diagnoses among ACSMs between 2018 and 2022. The prevalence of vitamin D deficiency among the active component was lower than that of the U.S. general population. This difference is likely due to methodology, as ICD-9 and ICD-10-coded diagnoses were used in this analysis. In contrast, the NHANES studies performed serum 25(OH)D measurements on samples of the U.S. population, finding a prevalence of 22-24% that varies by age, race, and ethnicity.^13,14,15^ The active component is not routinely screened for vitamin D deficiency,^20^ making symptomatic service members more likely to be tested. A cross-sectional study of hospitalized U.S. adults using ICD-10 codes to identify vitamin D prevalence found a rate of 1.8%,^21^ similar to the present study’s findings.

Demographic factors associated with vitamin D deficiency were consistent with findings reported in studies of the general U.S. population, except for age. In the general U.S. population, the largest proportion of vitamin D deficiency is seen in non-Hispanic Black individuals, followed by Hispanic and non-Hispanic White individuals.^13^ In this study, the largest proportion of vitamin D deficiency was seen in non-Hispanic Black ACSMs, those of unknown race and ethnicity, and Hispanic ACSMs. Previously described demographic risk factors, such as obesity,^3,15^ a history of malabsorption syndrome,^5,16^ residing at a latitude below 33°,^17,18^ and working indoors,^11^ are associated with vitamin D deficiency among ACSMs. Those with obesity may be at higher risk for vitamin D deficiency, as increased BMI has been shown to correlate with lower vitamin D3 levels due to vitamin D sequestering in body fat.^22^ Those with intestinal malabsorption syndromes have reduced uptake of fat-soluble vitamins such as vitamin D.^16^ At latitudes farther from the equator, the ozone layer absorbs more ultraviolet-B radiation (required for cutaneous vitamin D production).^17^

In the active component, the 30-39 years and 40 years or older age groups had higher odds of vitamin D deficiency compared to younger age groups, after controlling for covariates and occupation. This result may be partly due to transitioning to a supervisory role as rank increases^23^ or increased opportunity for testing and diagnosis of vitamin D deficiency due to more frequent health care contact.^24^ Higher rates of vitamin D deficiency in young adults in the general U.S. population may be due to increased time indoors.^25^ Obesity prevalence increases by age in the active component,^26^ but the higher odds of vitamin D deficiency remain for older members after adjusting for obesity. In those taking more frequent multivitamins and vitamin D supplementation, vitamin D deficiency was more common. This is potentially due to reverse causality, with members likely taking vitamin D supplements because they had been diagnosed with vitamin D deficiency.

In this study, women were more likely to be diagnosed with vitamin D deficiency. This finding may be in part due to increased testing compared to men, although women have been shown to have lower vitamin D levels in other studies.^13,14,27^ Despite our knowledge of the vital role that vitamin D plays in bone health, bone mineral density increases in women in their 30s, and women ages 65 years or older are at higher risk for osteoporotic fracture.^28,29,30^ It is unclear how vitamin D levels in early adulthood predict the risk of osteoporosis later in life. Calcium, and not vitamin D supplementation, has been shown to increase bone mineral density.^31^ The time horizon for a study to evaluate this association would be decades.

The higher rates of vitamin D deficiency seen in recruits compared to enlisted ACSMs, warrant officers, and officers may be due to surveillance bias. It is not uncommon for a recruit trainee to have a vitamin D level ordered when being evaluated for a stress fracture. Military training instructors had a prevalence similar to indoor workers, and this may be due to more time spent indoors instructing in a classroom than instructing outdoors. Unmanned vehicle operators had the lowest rates of all the occupation subgroups, and this may be due to training and occupational duties that require time outdoors. This may also be due to flexible schedules allowing on- and off-duty outdoor sunlight exposure or avoiding medical care and laboratory testing. It is important to note that pilots and air crew had the lowest odds of developing vitamin D deficiency of the primary occupational categories. In conjunction with the higher rates of melanoma in aviators,^32^ the lower rates of vitamin D deficiency are likely due to increased sunlight exposure in this occupation. Although we were unable to evaluate submariners in this study specifically, it is known that they do not receive ultraviolet-B exposure during patrol and are exposed to other factors that may affect bone health.^12^

In those who receive little sunlight exposure, supplementing vitamin D or consuming foods containing vitamin D may become essential to maintain adequate 25(OH) D serum levels. The recommended daily allowance for vitamin D in the general population is 600 IU daily.^33^ Still, higher levels may be needed for those without sunlight, such as submariners on patrol. A dosage of 1,000 IU daily has been proposed for submariners.^34^

There were some limitations to this study, which potentially included unmeasured confounding. The incidence and prevalence were likely underestimated compared to studies of the U.S. population using NHANES data due to the reliance on ICD-coded diagnosis data. It was impossible to capture off-duty sunlight exposure and sunscreen use, which may confound associations with other demographic risk factors such as age or occupation. PHA data are collected for patient-provider health assessments and decision-making and are not designed for epidemiologic surveillance, which led to the inability to establish temporality between dietary factors and vitamin supplementation with vitamin D deficiency. PHA dietary and vitamin data are self-reported, leading to misclassification bias and generating many unknown values from missing PHAs or non-responses.

Future studies may consider sampling ACSMs and performing serum 25(OH) D measurements via liquid chromatography coupled with tandem mass spectrometry (LC-MS/MS) for more accurate estimates of the incidence and prevalence of vitamin D deficiency in the active component. This will help inform future policies on screening and treatment. LC-MS/MS is considered the ‘gold standard’ for measuring 25(OH) D, as other assays have intrinsic analytical issues.^35^ Clinicians should consider individual risk factors for measuring vitamin D levels (e.g., persons other than non-Hispanic White individuals, having obesity or malabsorption syndrome, female sex, indoor occupation, and residing at a latitude above 33°), particularly if a service member gets little exposure to sunlight. It would be reasonable to allow targeted vitamin D screening in at-risk members. Additionally, education and ensuring adequate intake (600 IU daily) for all active component members is essential, with a particular focus on those at risk for vitamin D deficiency. Higher levels of vitamin D intake may be necessary in those with negligible to no sunlight exposure (i.e., submariners on patrol).

## Figures and Tables

**Figure:**
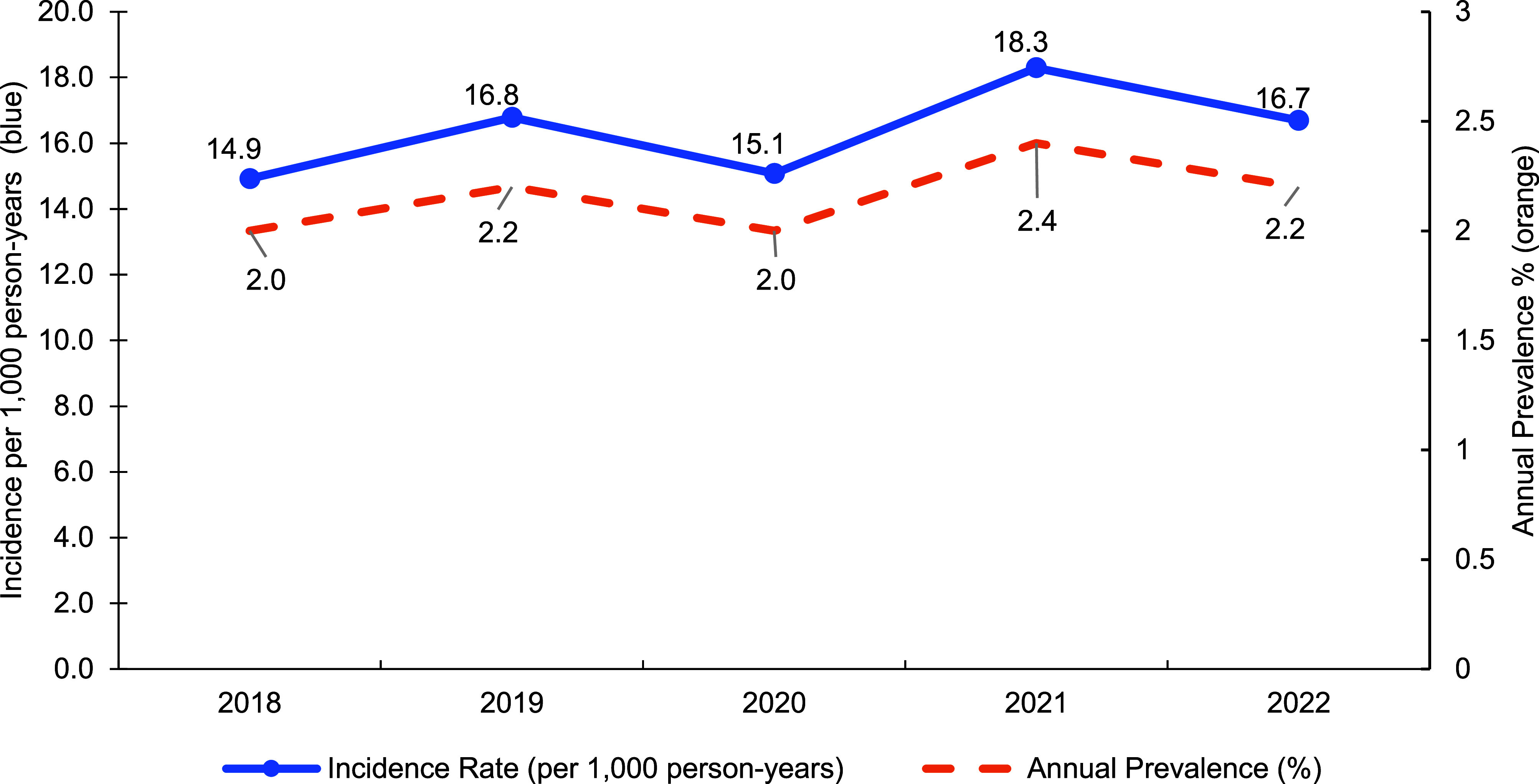
Vitamin D Deficiency Diagnoses, Active Component, 2018–2022

**Table 1:** Incidence Rate (per 1,000 person-years) of First Diagnosis and Total Average Annual Prevalence of Vitamin D Deficiency Diagnosis, Active Component, U.S. Armed Forces, 2018–2022

	Incidence		Average Annual Prevalence	
	No.	Rate	No.	%
Total	104,994	16.4	146,753	2.2
Sex				
Male	69,562	12.9	94,009	1.7
Female	35,432	34.4	52,744	4.6
Age group, y				
<20	6,114	13.1	6,499	1.5
20–29	45,494	12.5	56,970	1.5
30–39	33,807	19.2	49,915	2.6
40+	19,579	35.6	33,369	5.2
Race and ethnicity				
White, non-Hispanic	47,096	13.2	64,023	1.7
Hispanic	18,741	16.9	25,685	2.2
Black, non-Hispanic	26,872	27.1	39,764	3.7
Other	10,254	15.6	14,158	2.0
Unknown	2,031	19.7	3,123	2.8
Military rank				
Recruit	4,900	37.2	4,935	4.5
Enlisted (non-recruit)	81,789	15.8	113,204	2.1
Warrant officer	1,866	21.7	2,889	3.1
Officer	16,439	15.8	25,725	2.3
Branch of service				
Army	44,693	19.3	63,808	2.6
Navy	22,955	14	30,371	1.8
Air Force	30,289	19.4	43,611	2.6
Marine Corps	7,057	7.8	8,963	1.0
Latitude of military unit				
>33 degrees	68,868	18	96,423	2.4
<=33 degrees	35,509	14	49,446	1.9
Unknown	617	12.7	884	1.6
Obese				
No	81,464	14.2	111,406	1.9
Yes	23,530	35.4	35,347	4.7
Malabsorption syndrome				
No	103,132	16.2	143,448	2.1
Yes	1,862	58.1	3,305	8.2
Multivitamin supplementation				
<=Once a week	58,629	15.3	80,669	2.0
>Once a week	34,822	19.8	51,270	2.7
Unknown	11,543	13.9	14,814	1.8
Dairy and calcium-containing foods				
<2 servings per day	76,875	17.1	108,887	2.3
>=2 servings per day	16,576	15.1	23,052	2.0
Unknown	11,543	13.9	14,814	1.8
Fish consumption				
<1 serving per day	85,168	16.9	120,479	2.2
>=1 serving per day	8,283	15	11,460	2.0
Unknown	11,543	13.9	14,814	1.8
Primary occupational category				
Combat-specific	10,216	11.6	13,766	1.5
Motor transport	3,893	18.2	4,756	2.2
Pilot/air crew	1,313	6.4	1,714	0.8
Repair/engineering	22,124	12.1	29,387	1.5
Communications/intelligence	26,867	20.7	39,645	2.8
Health care	14,315	30	22,516	4.2
Other	26,266	17.3	34,969	2.2
Occupation group				
Indoor	23,685	27	36,605	3.7
Outdoor	10,669	13.1	14,206	1.7
All other	70,640	14.9	95,942	1.9
Occupation subgroup				
Indoor: administrative and legal	7,274	26.4	11,341	3.7
Indoor: unmanned vehicle operators	85	7.6	109	0.9
Indoor: non-medical scientists, mathematicians	433	24.7	675	3.5
Indoor: chaplains and assistants	653	26.4	1,014	3.7
Indoor: health care worker	12,763	29.7	19,967	4.1
Indoor: veterinarian services	306	34.5	467	4.6
Indoor: food service and sales	2,171	20.2	3,032	2.6
Outdoor: combat professions	4,846	10.8	6,328	1.4
Outdoor: EOD/UDT/divers	337	12.1	470	1.6
Outdoor: security/firefighters	4,110	14.7	5,360	1.8
Outdoor: military training instructor	1,376	23.8	2,048	3.3
All other	70,640	14.9	95,942	1.9

Abbreviations: No., number; y, years; EOD, explosive and ordnance disposal; UDT, underwater demolition team.

**Table 2:** Adjusted Odds Ratios for Vitamin D Deficiency Diagnosis, Active Component Service Members, 2022

	aOR	95% LL	95% UL
Sex				
Male	Ref	--	--
Female	2.4	2.3	2.5
Age group, y				
<20	Ref	--	--
20–29	0.9	0.8	0.9
30–39	1.4	1.3	1.4
40+	2.6	2.5	2.8
Race and ethnicity				
White, non-Hispanic	Ref	--	--
Hispanic	1.4	1.3	1.4
Black, non-Hispanic	1.8	1.7	1.8
Other	1.1	1.0	1.1
Unknown	1.4	1.3	1.5
Branch of service				
Marine Corps	Ref	--	--
Army	1.9	1.8	2.0
Navy	1.5	1.4	1.6
Air Force	1.6	1.5	1.7
Latitude of military unit				
<=33 degree	Ref	--	--
>33 degree	1.4	1.4	1.5
Unknown	1.4	1.2	1.6
Obesity				
No	Ref	--	--
Yes	2.2	2.2	2.3
Malabsorption syndrome				
No	Ref	--	--
Yes	2.5	2.3	2.7
Primary occupational category				
Combat-specific	Ref	--	--
Motor transport	1.2	1.1	1.3
Pilot/air crew	0.5	0.3	0.6
Repair/engineering	1.0	0.9	1.0
Communications/intelligence	1.3	1.2	1.4
Health care	1.6	1.5	1.6
Other	1.3	1.2	1.3

Abbreviations: aOR, adjusted odds ratio; LL, lower limit; UL, upper limit; Ref, reference; y, years.

## AUTHOR AFFILIATIONS

Uniformed Services University of the Health Sciences, Department of Preventive Medicine and Biostatistics, Bethesda, MD: Maj Kelly; Armed Forces Health Surveillance Division, Defense Health Agency, Silver Spring, MD: Dr. Fan, CAPT Langton, Dr. Stahlman

## DISCLAIMER

The opinions and assertions expressed herein are those of the authors and do not reflect the official policy nor position of the Uniformed Services University of the Health Sciences or the Department of Defense. This work was prepared by a military or civilian employee of the U.S. Government as part of official duties and therefore is in the public domain and does not possess copyright protection. Public domain information may be freely distributed and copied; as a courtesy, it is requested that the Uniformed Services University and author are appropriately acknowledged.

## References

[1] Pludowski P, Holick MF, Pilz S (2013). Vitamin D effects on musculoskeletal health, immunity, autoimmunity, cardiovascular disease, cancer, fertility, pregnancy, dementia and mortality–a review of recent evidence.. Autoimmun Rev..

[2] National Institute of Diabetes and Digestive and Kidney Diseases. LiverTox: Clinical and Research Information on Drug-Induced Liver Injury..

[3] Holick MF, Binkley NC, Bischoff-Ferrari HA (2011). Evaluation, treatment, and prevention of vitamin D deficiency: an Endocrine Society clinical practice guideline.. J Clin Endocrinol Metab..

[4] Yoon S, Kwon O, Kim J (2021). Vitamin D in athletes: focus on physical performance and musculoskeletal injuries.. Phys Act Nutr..

[5] Holick MF (2007). Vitamin D deficiency.. NEJM..

[6] Martineau AR, Jolliffe DA, Hooper RL (2017). Vitamin D supplementation to prevent acute respiratory tract infections: systematic review and meta-analysis of individual participant data.. BMJ..

[7] Burgi AA, Gorham ED, Garland CF (2011). High serum 25-hydroxyvitamin D is associated with a low incidence of stress fractures.. J Bone Miner Res..

[8] Davey T, Lanham-New SA, Shaw AM (2016). Low serum 25-hydroxyvitamin D is associated with increased risk of stress fracture during Royal Marine recruit training.. Osteoporos Int..

[9] Dao D, Sodhi S, Tabasinejad R (2015). Serum 25-hydroxyvitamin D levels and stress fractures in military personnel: a systematic review and meta analysis.. Am J Sports Med..

[10] Lappe J, Cullen D, Haynatzki G (2008). Calcium and vitamin D supplementation decreases incidence of stress fractures in female navy recruits.. J Bone Miner Res..

[11] Sowah D, Fan X, Dennett L, Hagtvedt R, Straube S (2017). Vitamin D levels and deficiency with different occupations: a systematic review.. BMC Public Health..

[12] Henriques M, Rodrigues D, Viegas S, Serranheira F, Sacadura-Leite E (2023). Vitamin D status in active duty Navy military personnel: a systematic review.. Occup Environ Med..

[13] Schleicher RL, Sternberg MR, Looker AC (2016). National estimates of serum total 25-hydroxyvitamin D and metabolite concentrations measured by liquid chromatography–tandem mass spectrometry in the US population during 2007–2010.. J Nutr..

[14] Herrick KA, Storandt RJ, Afful J (2019). Vitamin D status in the United States, 2011-2014.. Am J Clin Nutr..

[15] Cui A, Xiao P, Ma Y (2022). Prevalence, trend, and predictor analyses of vitamin D deficiency in the US population, 2001-2018.. Front Nutr..

[16] Margulies SL, Kurian D, Elliott MS, Han Z (2015). Vitamin D deficiency in patients with intestinal malabsorption syndromes–think in and outside the gut.. J Dig Dis..

[17] Wacker M, Holick MF (2013). Sunlight and vitamin D: a global perspective for health.. Dermatoendocrinol..

[18] Leary PF, Zamfirova I, Au J, McCracken WH (2017). Effect of latitude on vitamin D levels.. J Am Osteopath Assoc..

[19] Dairy.. MyPlate..

[20] Defense Health Agency Vitamin D Screening.. TRICARE..

[21] Patel U, Yousuf S, Lakhani K (2020). Prevalence and outcomes associated with vitamin D deficiency among indexed hospitalizations with cardiovascular disease and cerebrovascular disorder–a nationwide study.. Medicines (Basel)..

[22] Wortsman J, Matsuoka LY, Chen TC, Lu Z, Holick MF (2000). Decreased bioavailability of vitamin D in obesity.. Am J Clin Nutr..

[23] Office of the Deputy Assistant Secretary of Defense for Military Community and Family Policy., ICF. 2020 Demographics: Profile of the Military Community..

[24] Meadows SO, Engel CC, Collins RL (2021). 2018 Department of Defense Health Related Behaviors Survey (HRBS): Results for the Active Component..

[25] Tangpricha V, Pearce EN, Chen TC, Holick MF (2002). Vitamin D insufficiency among free-living healthy young adults.. Am J Med..

[26] Legg M, Stahlman S, Chauhan A (2022). Obesity prevalence among active component service members prior to and during the COVID-19 pandemic, January 2018-July 2021.. MSMR..

[27] Wierzbicka A, Oczkowicz M (2022). Sex differences in vitamin D metabolism, serum levels and action.. Br J Nutr..

[28] Recker RR, Davies KM, Hinders SM (1992). Bone gain in young adult women.. JAMA..

[29] Cawthon PM (2011). Gender differences in osteoporosis and fractures.. Clin Orthop Relat Res..

[30] Jiang X, Westermann LB, Galleo GV (2013). Age as a predictor of osteoporotic fracture compared with current risk-prediction models.. Obstet Gynecol..

[31] Voulgaridou G, Papadopoulou SK, Detopoulou P (2023). Vitamin D and calcium in osteoporosis, and the role of bone turnover markers: a narrative review of recent data from RCTs.. Diseases..

[32] U.S. Department of Defense. (2022). Phase 1-a-Study on the Incidence of Cancer Diagnosis and Mortality Among Military Aviators and Aviation Support Personnel..

[33] Demay MB, Pittas AG, Bikle DD (2024). Vitamin D for the prevention of disease: an Endocrine Society clinical practice guideline.. J Clin Endocrinol Metab..

[34] Gertner J, Horn W (2008). Vitamin D Supplementation in Submariners..

[35] Zelzer S, Goessler W, Herrmann M (2018). Measurement of vitamin D metabolites by mass spectrometry, an analytical challenge.. J Lab Precision Med..

